# Dr. George Michael Edebohls

**Published:** 1908-09

**Authors:** 


### OBITUARY

Dr. George Michael Edebohls, of New York, died at his home in that city August 8, 1908, from Hodgkin's disease after an illness of four months, aged 55 years. Dr. Edebohls graduated in medicine from the College of Physicians and Surgeons, now the medical department of Columbia University, in 1875. He became distinguished for his gynecological work, and rose to prominence in several medical societies. At the time of his death he was professor of diseases of women at the New York Post-Graduate Medical School and Hospital; attending gynecologist to Saint Francis and the Post-Graduate Hospitals; and consulting gynecologist to Saint Johns's Hospital, Yonkers, and to Nyack Hospital. He attained fame as an abdominal surgeon, his most conspicuous recent achievement being the operation of decapsulation of the kidney for the relief of nephritis.

